# Impact of an Antimicrobial Stewardship Program on Antibiotic Consumption, Bacterial Susceptibility, and Costs in a High-Complexity Public Hospital

**DOI:** 10.3390/antibiotics15030264

**Published:** 2026-03-03

**Authors:** Jéssica Cristina Bilizario Noguerol Andrade, Beatriz Souza Santos, Fernando de Sá Del Fiol

**Affiliations:** Doctoral Program, Pharmaceutical Sciences, University of Sorocaba, Sorocaba 18023-000, SP, Brazil; jessica.andrade@prof.uniso.br (J.C.B.N.A.);

**Keywords:** antimicrobial stewardship, anti-bacterial agents, drug resistance, bacterial, intensive care units, cost savings

## Abstract

**Background/Objectives**: Antimicrobial resistance (AMR) is a major global public health threat, particularly in hospitals. Antimicrobial Stewardship Programs (ASPs) aim to optimize prescribing, reduce unnecessary exposure to broad-spectrum agents, and mitigate resistance. This study evaluated the clinical, ecological, and economic impact of an ASP implemented in January 2021 in a high-complexity hospital in Brazil, focusing on antimicrobial consumption, temporal trends in bacterial susceptibility, and direct antimicrobial-related costs. **Methods**: A quasi-experimental pre–post study using an interrupted time-series design was conducted in the adult intensive care unit from January 2019 to December 2023. Antimicrobial consumption was measured as Defined Daily Doses per 1000 patient-days (DDD/1000-PD) for ceftriaxone, meropenem, piperacillin–tazobactam, vancomycin, and polymyxin B. Temporal trends were assessed using Joinpoint regression, and pre- and post-intervention periods were compared using Student’s or Mann–Whitney tests. Susceptibility data were interpreted according to BrCAST standards. **Results**: Significant and sustained reductions were observed for all agents except polymyxin B. Susceptibility improved or stabilized among key Gram-negative pathogens, with a significant increase in aggregated Gram-negative susceptibility after 2021, while intrinsically resistant organisms showed limited change. Annual antimicrobial costs decreased by approximately USD 174,000. **Conclusions**: The ASP was associated with reduced broad-spectrum antimicrobial use, favorable ecological trends, and substantial cost savings.

## 1. Introduction

Antimicrobial resistance (AMR) is widely recognized as one of the most serious global threats to health, food security, and development and has been described as a “silent pandemic”. The spread of resistant microorganisms compromises the effectiveness of available treatments, increases healthcare costs, and leads to higher morbidity and mortality from infections that were previously controllable. Global agencies, including the World Health Organization (WHO) and the Organisation for Economic Co-operation and Development (OECD), have warned that without coordinated and evidence-based action, AMR may reverse decades of medical progress and impose substantial human and economic losses worldwide [1,2,3,4]. In hospitals, this problem is amplified by the concentration of vulnerable patients, frequent invasive procedures, and intensive use of broad-spectrum antimicrobials, particularly in low- and middle-income countries where diagnostic and surveillance capacities are often limited [4,5].

Antimicrobial Stewardship Programs (ASPs) have emerged as a central strategy to optimize antimicrobial use, improve clinical outcomes, and curb the development of resistance. ASPs consist of coordinated interventions to ensure the appropriate selection, dosing, route, and duration of antimicrobial therapy based on patient characteristics and local epidemiology. Core components include institutional commitment, defined leadership, audit and feedback, formulary restrictions, and continuous prescriber education [6,7,8]. Evidence from systematic reviews and meta-analyses demonstrates that ASPs reduce unnecessary antimicrobial exposure, particularly to broad-spectrum agents, decrease rates of multidrug-resistant organisms and Clostridioides difficile infection, and generate substantial cost savings without compromising patient safety [3,9,10]. Moreover, stewardship promotes cultural change within institutions by fostering multidisciplinary collaboration and sustained improvements in prescribing behavior [11,12].

### Brazilian Context and Remaining Gaps

In Brazil, AMR has been intensified by longstanding patterns of antibiotic overuse, self-medication, and previously unregulated access, contributing to the spread of multidrug-resistant organisms such as carbapenem-resistant *Klebsiella pneumoniae* and *Acinetobacter* spp. Although the Brazilian Health Regulatory Agency (ANVISA) has aligned national surveillance and stewardship policies with WHO recommendations and published national guidelines for ASP implementation [13], adoption remains heterogeneous and often limited by shortages of trained personnel, insufficient microbiological infrastructure, and persistent cultural barriers to rational prescribing [14]. Standardized metrics such as Defined Daily Doses per 1000 patient-days and systematic susceptibility surveillance are essential to evaluate and guide stewardship activities, particularly in high-risk environments such as intensive care units [8,15,16,17].

Within this national context, a high-complexity public hospital in the State of São Paulo implemented a structured ASP in January 2021, incorporating audit and feedback, protocol development, microbiological surveillance, and systematic monitoring of antimicrobial consumption. The objective of this study was to assess the impact of the Antimicrobial Stewardship Program (ASP) implemented in January 2021 on three domains: (1) antimicrobial consumption measured in DDD per 1000 patient-days, (2) temporal trends in bacterial susceptibility as a proxy for changes in the hospital eclogy, and (3) direct antimicrobial-related costs.

## 2. Results

Table 1 shows the distribution of the most frequently isolated microorganisms, with *K. pneumoniae*, *Staphylococcus aureus*, and *Escherichia coli* representing the predominant species in the dataset. This pattern is consistent with international epidemiological reports indicating these pathogens as leading causes of healthcare-associated infections in critically ill patients [18,19]. Notably, several organisms within the ESKAPE group: *Enterococcus faecium*, *Staphylococcus aureus*, *Klebsiella pneumoniae*, *Acinetobacter baumannii*, *Pseudomonas aeruginosa*, and *Enterobacter* spp. were among the most prevalent, reflecting their well-documented ability to thrive in hospital environments and contribute substantially to the global burden of difficult-to-treat infections [20]. The persistent predominance of these species reinforces longstanding concerns raised in the literature regarding their clinical relevance and adaptive capacity, underscoring the importance of continuous microbiological surveillance and robust antimicrobial stewardship initiatives [21].

### 2.1. Antimicrobial Consumption

#### 2.1.1. Ceftriaxone (Figure 1a,b)

**Figure 1:**
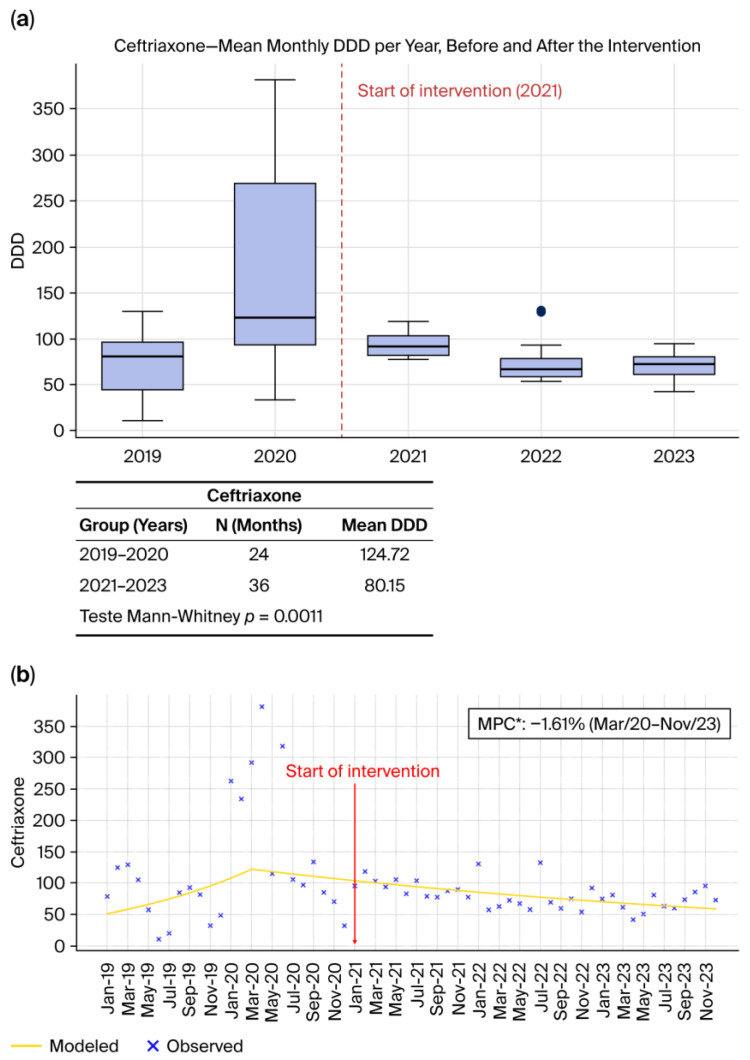
(**a**) Monthly mean DDD per year before and after the intervention. (**b**) Observed and Joinpoint-modeled consumption trends, showing the change in trajectory following the intervention point. * (*p* < 0.05).

Ceftriaxone consumption showed a marked and statistically significant reduction following the implementation of the Antimicrobial Stewardship Program (ASP). As illustrated in Figure 1a, the DDD/1000 patient-days decreased substantially in the post-intervention period, a difference confirmed by the Mann–Whitney test (*p* = 0.0011). Joinpoint regression analysis (Figure 1b) further confirms this trend, revealing that from March 2020 to November 2023 ceftriaxone consumption declined at a rate of 1.61% per month (*p* < 0.05), shifting from relative stability or upward movement to a declining trajectory after 2021. Similar declines have been consistently reported in the literature, particularly in settings where stewardship interventions incorporate prospective audit and feedback, formulary oversight, and strengthened diagnostic pathways, as described by Bhattacharjee et al. (2025) [6] and in the IDSA/SHEA guidelines [8]. These findings reinforce established evidence that third-generation cephalosporins are among the antimicrobials most responsive to stewardship activities targeting empiric overuse.

#### 2.1.2. Meropenem (Figure 2a,b)

**Figure 2:**
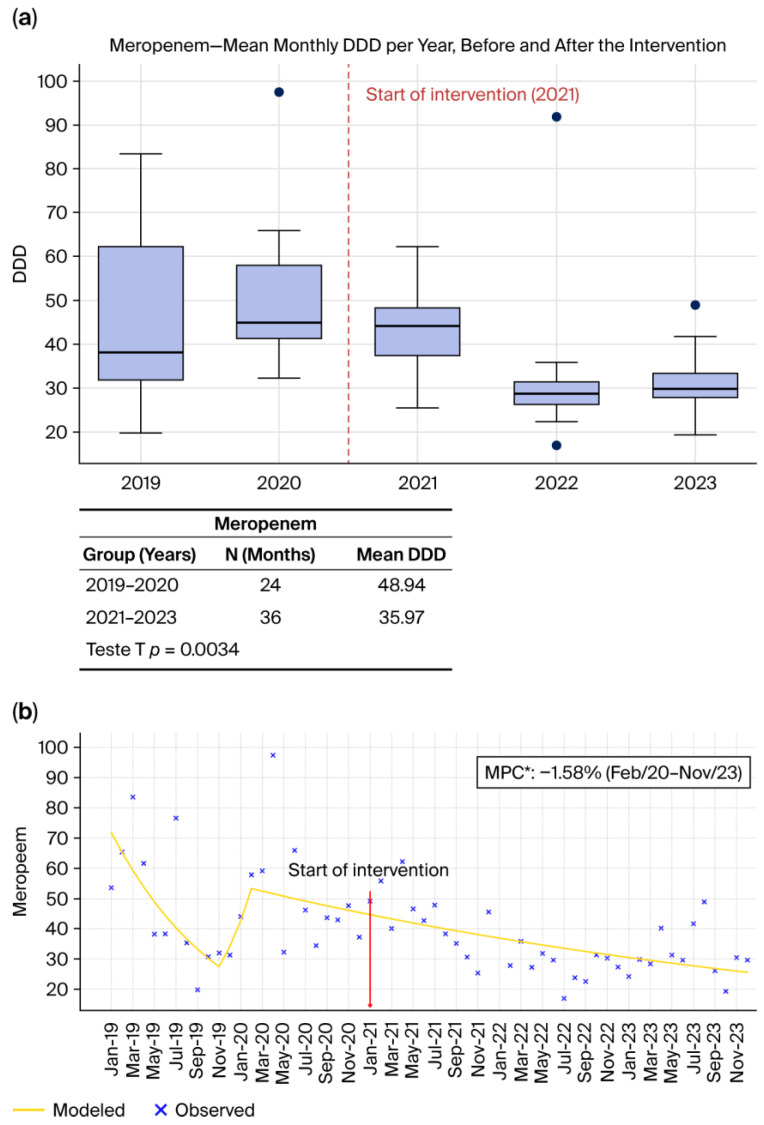
(**a**) Monthly mean DDD per year before and after the intervention. (**b**) Observed and Joinpoint-modeled consumption trends, showing the change in trajectory following the intervention point. * (*p* < 0.05).

Meropenem exhibited one of the most pronounced reductions among the evaluated agents. Figure 2a shows a significant decrease in annual consumption after the intervention, supported by a statistically significant difference in the *t*-test (*p* = 0.0034). The Joinpoint regression analysis (Figure 2b) further characterizes this pattern, revealing that from March 2020 to November 2023 meropenem consumption decreased at a rate of −1.58% per month (*p* < 0.05), marking a sustained reversal of the pre-intervention upward trend. Similar declines have been consistently reported in international assessments, including OECD analyses (2018) [3] and by Fattahniya et al. (2025) [9], which demonstrate that structured stewardship programs reliably decrease carbapenem consumption, an essential outcome given the central role of carbapenems in selecting resistant Gram-negative pathogens. The decline observed here aligns with the broader evidence base showing that carbapenem stewardship is a cornerstone of resistance mitigation strategies in high-complexity hospitals.

#### 2.1.3. Piperacillin–Tazobactam (Figure 3a,b)

**Figure 3:**
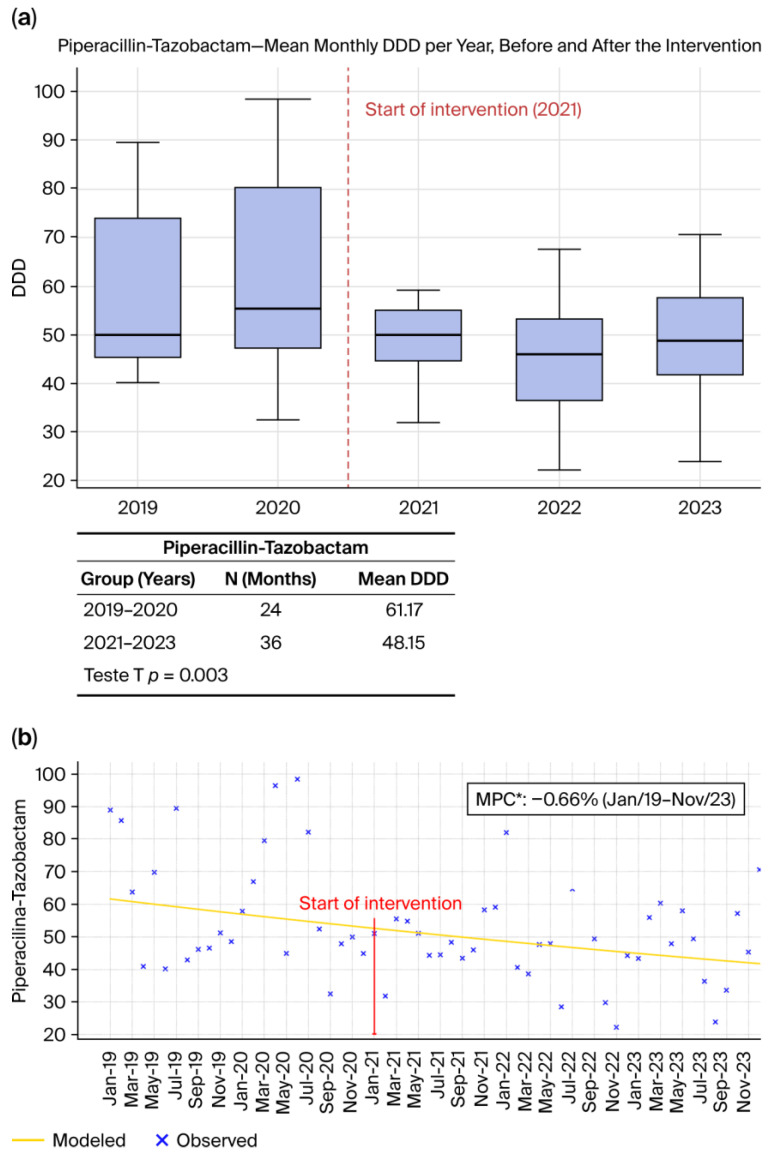
(**a**) Monthly mean DDD per year before and after the intervention. (**b**) Observed and Joinpoint-modeled consumption trends, showing the change in trajectory following the intervention point. * (*p* < 0.05).

Piperacillin–tazobactam consumption also declined significantly after ASP initiation. As shown in Figure 3a, DDD/1000 patient-days values were lower in the post-intervention period, and this reduction was statistically validated by the *t*-test (*p* = 0.003). Joinpoint regression (Figure 3b) further confirms this continuous downward trajectory, demonstrating that piperacillin–tazobactam use decreased across the full observation period at a rate of −0.66% per month (*p* < 0.05). International data similarly indicate that stewardship interventions emphasizing diagnostic stewardship, de-escalation, and adherence to guideline-based empiric therapy lead to substantial reductions in antipseudomonal β-lactam use, as demonstrated by Ullman et al. (2013) [11] and Kyriazopoulou and Giamarellos-Bourboulis (2022) [17]. These results are consistent with global findings showing that piperacillin–tazobactam is particularly sensitive to improvements in clinical decision-making and microbiological feedback systems.

#### 2.1.4. Polymyxin B (Figure 4a,b)

**Figure 4:**
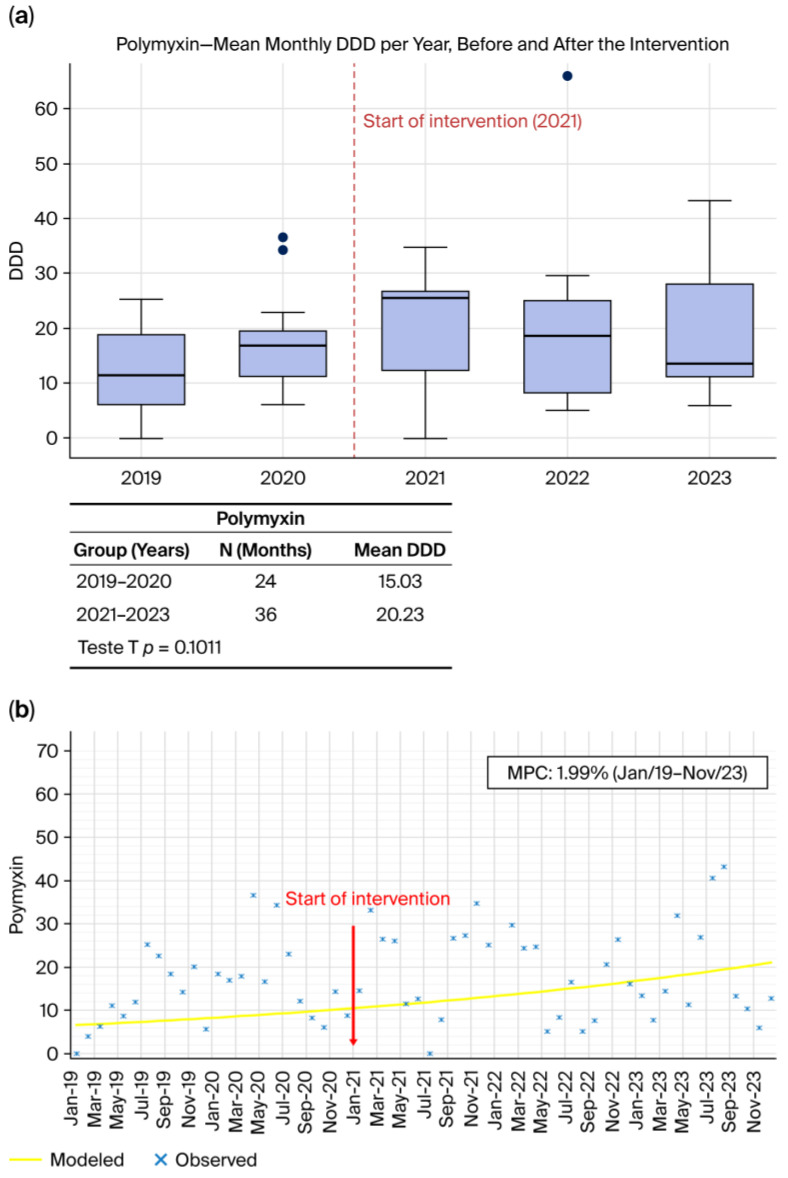
(**a**) Monthly mean DDD per year before and after the intervention. (**b**) Observed and Joinpoint-modeled consumption trends, showing the change in trajectory following the intervention point.

Polymyxin B consumption did not show a meaningful reduction following ASP implementation. As illustrated in Figure 4a, consumption in the pre-intervention period (2019–2020) was 15.03 DDD/1000 patient-days, compared with 20.23 DDD/1000 patient-days in the post-intervention period (2021–2023), reflecting a slight increase rather than improvement. This difference was not statistically significant (*p* = 0.1011). The Joinpoint analysis (Figure 4b) further demonstrates that throughout the entire study period, polymyxin B use followed a steady upward trend, increasing at a rate of 1.99% per month, although this rise did not reach statistical significance. No inflection point was detected, and the overall pattern indicates relative stability rather than a response to the intervention. These observations are consistent with global analyses by Laxminarayan et al. (2016) [4] and the OECD (2018) [3], which emphasize that reductions in last-resort agents such as polymyxins typically occur only after substantial and sustained declines in carbapenem-resistant Gram-negative infections. As noted by Fattahniya et al. (2025) [9], polymyxin consumption is primarily driven by the underlying epidemiology, and stewardship interventions alone rarely reduce its use unless resistance pressure simultaneously decreases.

#### 2.1.5. Vancomycin (Figure 5a,b)

**Figure 5:**
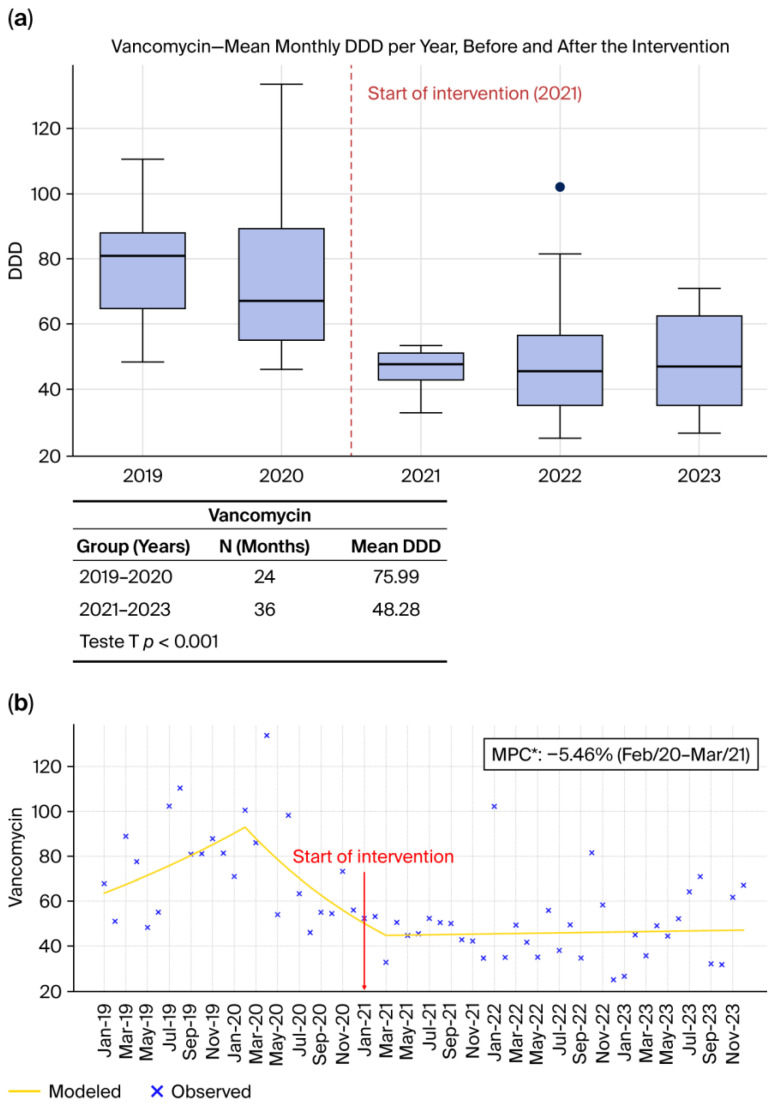
(**a**) Monthly mean DDD per year before and after the intervention. (**b**) Observed and Joinpoint-modeled consumption trends, showing the change in trajectory following the intervention point. * (*p* < 0.05).

Vancomycin (Figure 5a,b) consumption decreased substantially following ASP implementation. As shown in Figure 5a, the DDD/1000 patient-days declined from 75.99 in the pre-intervention period (2019–2020) to 48.28 in the post-intervention period (2021–2023), representing a marked reduction in glycopeptide use (*p* < 0.001). Importantly, the Joinpoint analysis (Figure 5b) identifies a statistically significant early downward segment: between February 2020 and March 2021, vancomycin consumption decreased at a rate of –5.46% per month (*p* < 0.05), indicating a rapid and meaningful decline even before the broader post-intervention sustained reduction. After this period, the temporal trajectory transitioned into a consistent downward pattern, reinforcing the overall impact of stewardship activities. These findings align with international literature showing that interventions emphasizing microbiological confirmation, biomarker-guided decision-making, and daily prescription review are highly effective in reducing unnecessary vancomycin use [8,17]. The magnitude of the decline observed here is consistent with global evidence showing that vancomycin prescribing is particularly responsive to systematic evaluation of MRSA risk, improved diagnostic turnaround times, and strengthened institutional prescribing policies.

Across all antibiotics evaluated, Figure 1a, Figure 2a, Figure 3a and Figure 4a, and Figure 5a consistently demonstrate that the interquartile ranges (IQRs) during the post-intervention years (2021, 2022, and 2023) became markedly narrower when compared with the pre-intervention period. This contraction of the quartile intervals indicates that monthly prescribing behavior became more uniform, with significantly less variability in antimicrobial consumption over time. Such stabilization is a well-recognized indicator of successful stewardship implementation, reflecting not only reductions in consumption but also improved prescribing discipline, greater adherence to rigid treatment protocols, and decreased reliance on discretionary or empiric antimicrobial use [8,22,23]. The narrowing of IQRs across multiple agents suggests that the ASP did not merely reduce overall antibiotic exposure but also standardized clinical practice, minimizing fluctuations associated with non-protocol-driven decisions. This pattern provides strong quantitative evidence that the structured stewardship strategies introduced after 2021, such as standardized protocols, prospective audit and feedback, and microbiology-guided prescribing, produced a sustained and system-wide improvement in prescribing consistency.

#### 2.1.6. Total J01 Antibacterial Consumption in the ICU

To evaluate whether reductions were limited to selected agents or reflected a broader ecological shift, total ICU antibacterial consumption at the ATC J01 level was analyzed, including all systemic antibacterials used during the study period (Amikacin, Ampicillin–sulbactam, Aztreonam, Cefepime, Cefotaxime, Ceftazidime, Ceftazidime–avibactam, Ceftolozane–tazobactam, Ceftriaxone, Ciprofloxacin [oral and parenteral], Ertapenem, Imipenem, Levofloxacin [oral and parenteral], Linezolid [oral and parenteral], Meropenem, Moxifloxacin [oral and parenteral], Piperacillin–tazobactam, Polymyxin B, Polymyxin E, Teicoplanin, Vancomycin, and Daptomycin).

Monthly aggregated J01 consumption (DDD/1000 patient-days) declined from a 410.24 DDD (SD 185.32) in the pre-intervention period (2019–2020) to 276.30 (SD 68.31) in the post-intervention period (2021–2023), corresponding to an absolute reduction of −133.94 DDD/1000 patient-days and a relative decrease of 32.65%. This difference was statistically significant (Welch’s *t*-test *p* = 0.0021; Mann–Whitney *p* = 0.00021; 95% CI −214.97 to −52.91).

A non-parametric temporal analysis confirmed a significant overall downward trend across the entire 2019–2023 period (Kendall’s tau = −0.358; *p* = 5.26 × 10^−5^). These findings demonstrate that the reduction in antimicrobial consumption after ASP implementation was not restricted to selected broad-spectrum agents but reflected a true decrease in total antibacterial exposure at the ICU level.

### 2.2. Effect of the Stewardship Program on the Hospital Ecology

#### 2.2.1. *Klebsiella pneumoniae*

**Figure 6:**
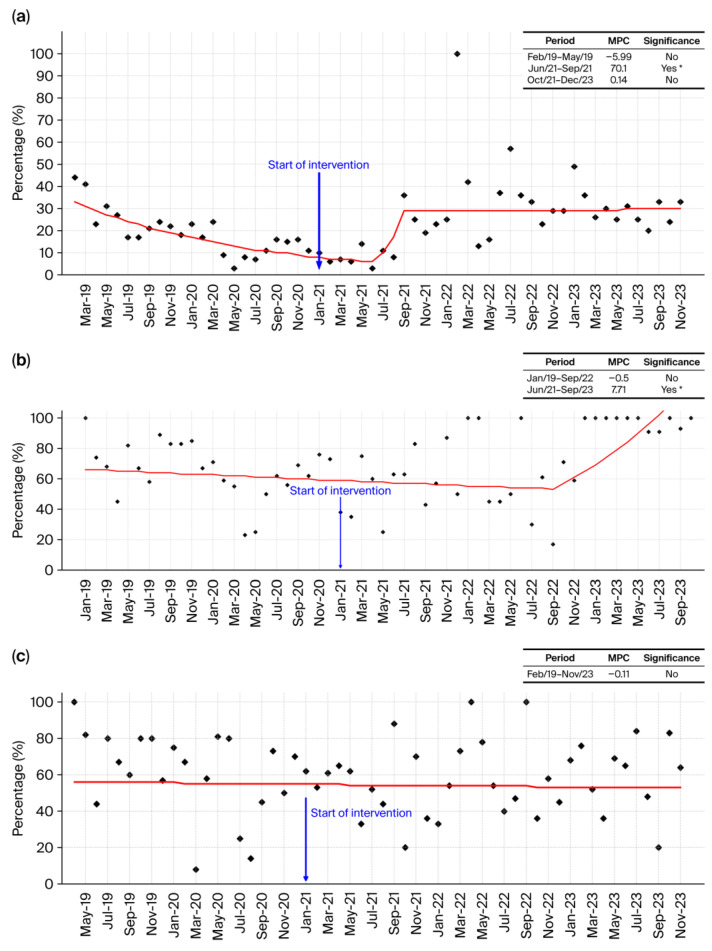

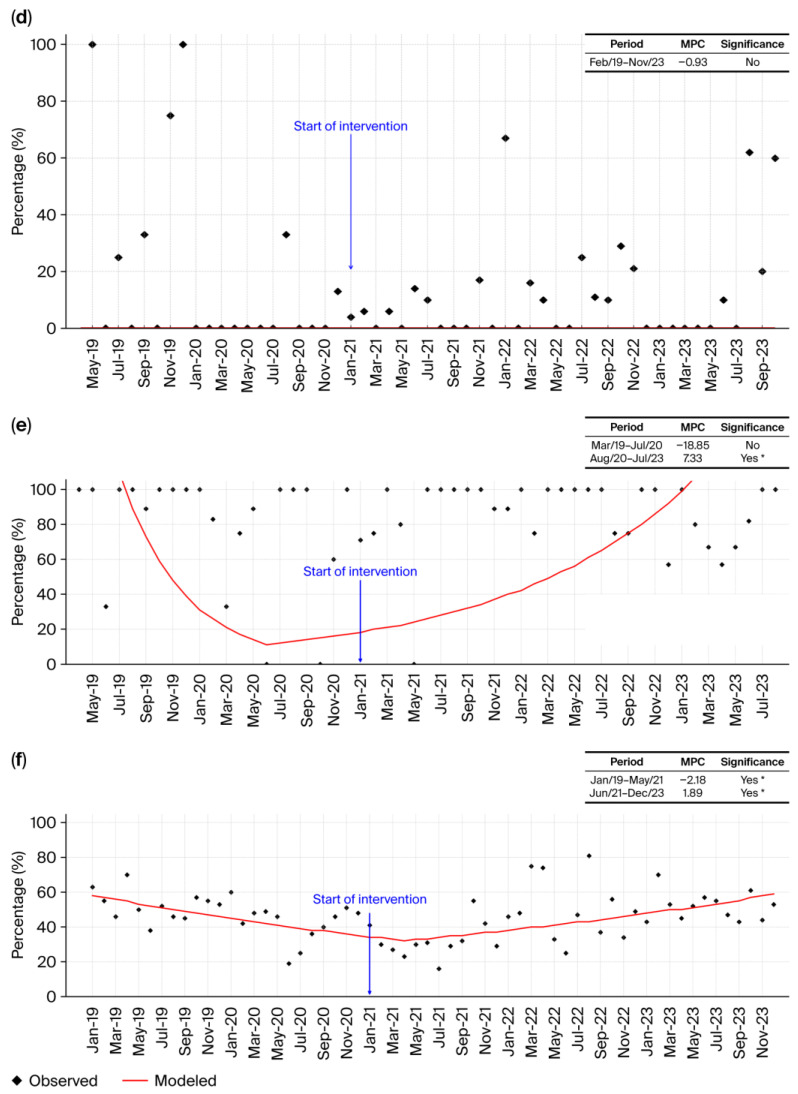
(**a**) Temporal trend in *Klebsiella pneumoniae* susceptibility (%) from February 2019 to December 2023. The table summarizes the Monthly Percentual Change (MPC) for each Joinpoint-identified segment, indicating periods of decline or improvement insusceptibility. Statistically significant MPC values (*p* < 0.05) are highlighted and reflect true changes in trend over time. (**b**) Temporal trend in *Escherichia coli* susceptibility (%) from January 2019 to September 2023. The table summarizes the Monthly Percentual Change (MPC) for each Joinpoint-identified segment, indicating periods of decline or improvement insusceptibility. Statistically significant MPC values (*p* < 0.05) are highlighted and reflect true changes in trend over time. (**c**) Temporal trend in *Pseudomonas aeruginosa* susceptibility (%) from February 2019 to November 2023. The table summarizes the Monthly Percentual Change (MPC) for each Joinpoint-identified segment, indicating periods of decline or improvement in susceptibility. Statistically significant MPC values (*p* < 0.05) are highlighted and reflect true changes in trend over time. (**d**) Temporal trend in *Acinetobacter baumanii* susceptibility (%) from February 2019 to November 2023. The table summarizes the Monthly Percentual Change (MPC) for each Joinpoint-identified segment, indicating periods of decline or improvement in susceptibility. Statistically significant MPC values (*p* < 0.05) are highlighted and reflect true changes in trend over time. (**e**) Temporal trend in *Enterobacter cloace* (%) from March 2019 to July 2023. The table summarizes the Monthly Percentual Change (MPC) for each Joinpoint-identified segment, indicating periods of decline or improvement in susceptibility. Statistically significant MPC values (*p* < 0.05) are highlighted and reflect true changes in trend overtime. (**f**) Temporal trend (%) from January 2019 to December 2023, representing all Gram-negative bacteria isolated in the study. The table summarizes the Monthly Percentual Change (MPC) for each Joinpoint-identified segment, indicating periods of decline or improvement in susceptibility. Statistically significant MPC values (*p* < 0.05) are highlighted and reflect true changes in trend over time. * Indicates that the MPC is significantly different from zero at the 0.05 alpha level.

*Klebsiella pneumoniae* (Figure 6a) demonstrated a pronounced temporal fluctuation in susceptibility throughout the study period, as depicted in Figure 6a. The early phase (2019–2020) showed a visually apparent decline in susceptibility, consistent with the first Joinpoint segment (February 2019–May 2019, MPC = −5.99%/month, non-significant). Although this decrease was not statistically significant, the downward pattern is reflected in the fitted trend line and persisted into late 2020, when susceptibility reached its lowest point. After the implementation of the Antimicrobial Stewardship Program (ASP) in January 2021, an upward shift became evident. The Joinpoint model detected a statistically significant improvement in the period June 2021–September 2021 (MPC = +70.1%/month, *p* < 0.05), indicating a rapid and clinically meaningful recovery in susceptibility. The subsequent segment (October 2021–December 2023) demonstrated stabilization at these improved levels (MPC = +0.14%/month, non-significant). While this stabilization is not statistically confirmed, the overall pattern suggests maintenance of the post-intervention gains.

This sequence: initial non-significant decline, followed by a statistically significant improvement shortly after ASP implementation, and subsequent stabilization—is consistent with global evidence that Gram-negative pathogens, particularly ESKAPE organisms such as *K. pneumoniae*, may exhibit shifts in susceptibility when antimicrobial pressure changes. Experimental findings by Daruka et al. (2025) [21] support the concept that *K. pneumoniae* can undergo rapid phenotypic adaptation in response to altered selective pressures. Taken together, the statistically significant improvement in 2021, combined with the visually stable trajectory afterward, suggests that the ASP may have contributed to the observed recovery in *K. pneumoniae* susceptibility, although non-significant segments should be interpreted with caution.

#### 2.2.2. *Escherichia coli*

*Escherichia coli* (Figure 6b) showed a predominantly stable susceptibility profile in the pre-intervention period, with a slight and non-significant downward trend from January 2019 to September 2022 (MPC = −0.5%/month), consistent with the flat trajectory observed in Figure 6b. Because this decline did not reach statistical significance, it should be interpreted as a visual pattern rather than a confirmed temporal trend.

Although susceptibility remained relatively unchanged immediately after the ASP was introduced in January 2021, a marked improvement emerged in the subsequent Joinpoint segment (June 2021–September 2023), where susceptibility increased significantly (MPC = +7.71%/month; *p* < 0.05). This statistically confirmed rise is visually evident in the upward curvature beginning in late 2022 and indicates a meaningful improvement in susceptibility over time.

While observational data do not allow causal inference, the timing and magnitude of this improvement may suggest that the ASP contributed to a progressive reduction in selective pressure on *E. coli*. Such a pattern aligns with international evidence indicating that stewardship interventions—particularly reductions in broad-spectrum β-lactams—can favorably influence resistance trajectories in Enterobacterales, as highlighted by Laxminarayan et al. (2016) [4] and Cassini et al. (2019) [19]. The significant upward shift therefore supports the interpretation that *E. coli* susceptibility benefited from the stewardship-associated changes in antimicrobial prescribing practices.

#### 2.2.3. *Pseudomonas aeruginosa*

*Pseudomonas aeruginosa* exhibited remarkable temporal stability in susceptibility throughout the study period, as illustrated in Figure 6c. The Joinpoint analysis identified only a single segment (February 2019–November 2023) with a negligible and non-significant decline (MPC = −0.11%/month), indicating an essentially flat trend with no meaningful improvement or deterioration over time. This stability persisted before and after the ASP implementation in January 2021, suggesting that stewardship measures did not substantially influence susceptibility in this species, an observation consistent with global reports that *P. aeruginosa* often displays intrinsic resistance mechanisms and variable epidemiological behavior that make rapid shifts less likely [4,19]. Overall, the temporal pattern indicates a species whose susceptibility profile remained largely unchanged during the intervention period.

#### 2.2.4. *Acinetobacter baumannii*

*Acinetobacter baumannii* displayed persistently low susceptibility throughout the entire study period, as shown in Figure 6d, with most monthly values clustered near zero and only occasional isolated increases. The Joinpoint analysis identified a single long segment (February 2019–November 2023) with a small, non-significant downward trend (MPC = −0.93%/month), indicating no meaningful improvement over time. This chronically low susceptibility pattern underscores the well-recognized therapeutic difficulty posed by *A. baumannii*, a pathogen characterized by extensive intrinsic resistance mechanisms and a high capacity for acquiring additional resistance determinants [24]. The near-constant low susceptibility observed reinforces its status as one of the most difficult Gram-negative organisms to manage in hospital settings.

#### 2.2.5. *Enterobacter cloacae*

*Enterobacter cloacae* exhibited a striking U-shaped temporal pattern in susceptibility, with an initial precipitous decline from March 2019 to July 2020 (MPC = −18.85%/month, non-significant), reaching its lowest point immediately before the ASP was implemented. Following the intervention in January 2021, a progressive and sustained improvement became evident, culminating in a statistically significant upward trend during the August 2020–July 2023 segment (MPC = +7.33%/month; *p* < 0.05). This recovery is clearly visible in Figure 6e, where the fitted trend line rises steadily after the intervention point, eventually returning to high susceptibility levels. Such a pattern strongly suggests that the ASP contributed to reducing selective pressure and improving antimicrobial effectiveness against this organism. Overall, the significant post-intervention improvement indicates a meaningful impact of stewardship activities on restoring *E. cloacae* susceptibility.

#### 2.2.6. All Gram-Negative Bacteria

When evaluated collectively, the Gram-negative pathogens demonstrated a coherent temporal pattern marked by an initial decline in susceptibility, followed by a progressive and sustained recovery after the introduction of the ASP in January 2021. As illustrated in the aggregated trend (Figure 6f), susceptibility decreased steadily from 2019 through late 2020, reaching its lowest point just before the intervention. After this inflection, the curve shows a clear upward trajectory (MPC = +1.89%/month; *p* < 0.05), with successive improvements extending through 2022 and 2023, indicating a broad-based restoration of antimicrobial effectiveness across species. The consistency of this recovery observed in Enterobacterales such as *K. pneumoniae* and *E. coli*, as well as in more challenging organisms like *Enterobacter cloacae* suggests that the intervention exerted a system-wide impact rather than isolated pathogen-specific effects. The reorganization of prescribing practices, stricter adherence to therapeutic protocols, and more frequent reliance on microbiological data likely contributed to reducing selective pressure and promoting a gradual rebound in susceptibility. Taken together, the synchronized improvement among all Gram-negative organisms strongly supports the conclusion that the ASP played a central role in reversing the declining susceptibility trends observed before 2021 and in establishing a new, more favorable equilibrium in the hospital’s microbiological landscape.

#### 2.2.7. *Staphylococcus aureus*

**Figure 7:**
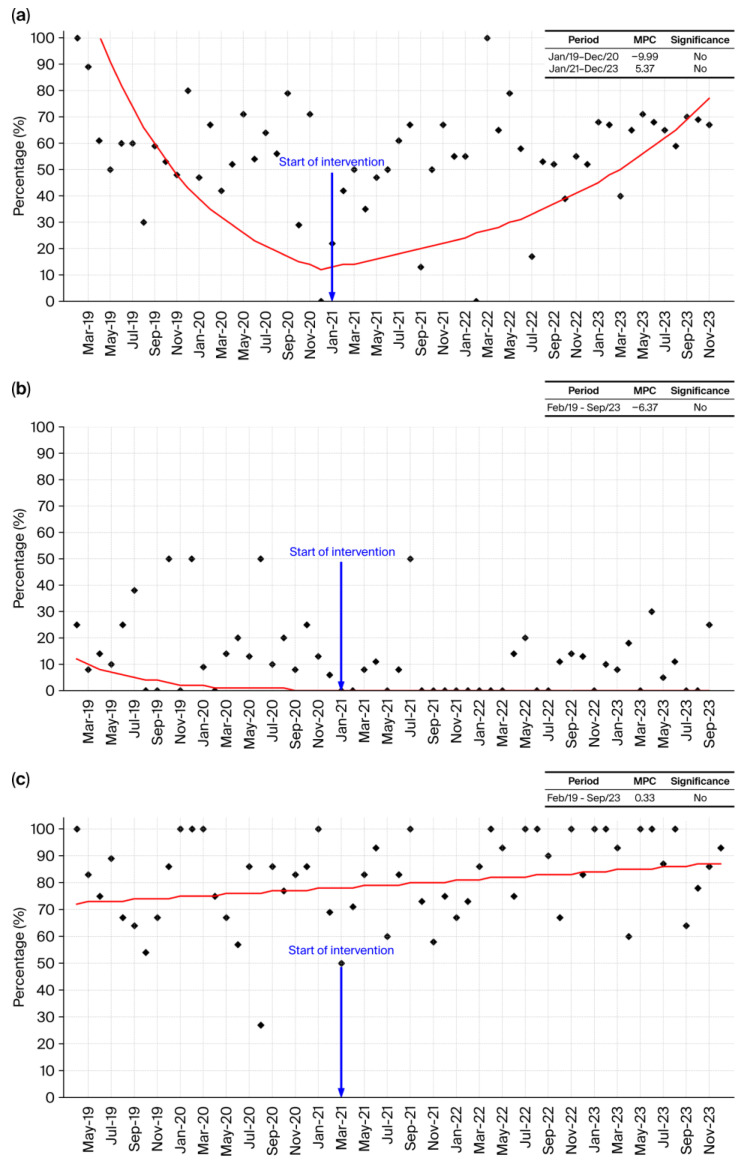

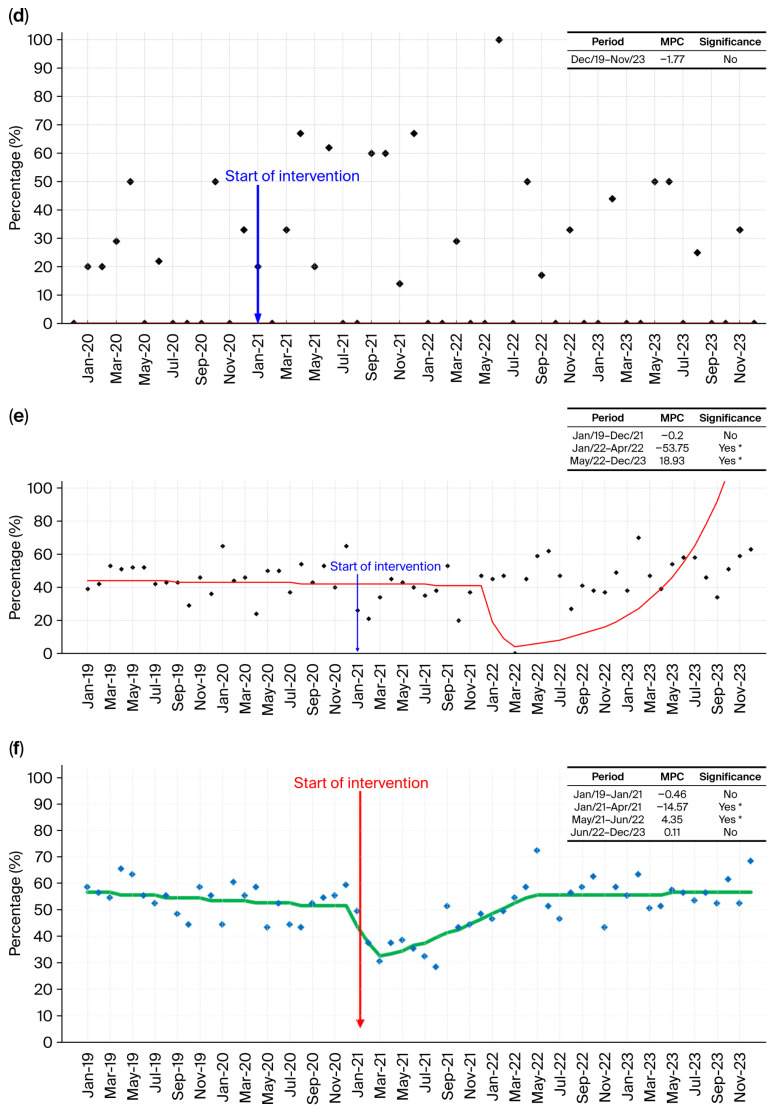
(**a**) Temporal trend in *Staphylococcus aureus* (%) from January 2019 to December 2023. The table summarizes the Monthly Percentual Change (MPC) for each Joinpoint-identified segment, indicating periods of decline or improvement in susceptibility. Statistically significant MPC values (*p* < 0.05) are highlighted and reflect true changes in trend over time. (**b**) Temporal trend in *Staphylococcus epidermidis* (%) from February 2019 to September 2023. The table summarizes the Monthly Percentual Change (MPC) for each Joinpoint-identified segment, indicating periods of decline or improvement insusceptibility. Statistically significant MPC values (*p* < 0.05) are highlighted and reflect true changes in trend over time. (**c**) Temporal trend in *Enterococcus faecalis* (%) from February 2019 to September 2023. The table summarizes the Monthly Percentual Change (MPC) for each Joinpoint-identified segment, indicating periods of decline or improvement in susceptibility. Statistically significant MPC values (*p* < 0.05) are highlighted and reflect true changes in trend over time. (**d**) Temporal trend in *Enterococcus faecium* (%) from December 2019 to November 2023. The table summarizes the Monthly Percentual Change (MPC) for each Joinpoint-identified segment, indicating periods of decline or improvement in susceptibility. Statistically significant MPC values (*p* < 0.05) are highlighted and reflect true changes in trend over time. (**e**) Temporal trend in all Gram-positive bacteria (%) from January 2019 to December 2023. The table summarizes the Monthly Percentual Change (MPC) for each Joinpoint-identified segment, indicating periods of decline or improvement in susceptibility. Statistically significant MPC values (*p* < 0.05) are highlighted and reflect true changes in trend over time. (**f**) Temporal trend for all tested bacteria (%) from January 2019 to December 2023. The table summarizes the Monthly Percentual Change (MPC) for each Joinpoint-identified segment, indicating periods of decline or improvement in susceptibility. Statistically significant MPC values (*p* < 0.05) are highlighted and reflect true changes in trend over time. * Indicates a statistically significant monthly percent change (*p* < 0.05). Dots represent the observed monthly values; the lines represent the trend estimated by Joinpoint regression.

*Staphylococcus aureus* (Figure 7a) displayed a V-shaped temporal pattern in susceptibility, although no Joinpoint-identified segment reached statistical significance. As illustrated in Figure 7a, susceptibility declined from January 2019 to December 2020 (MPC = −9.99%/month), followed by a gradual upward tendency after the ASP implementation in January 2021, extending through December 2023 (MPC = +5.37%/month; non-significant). While these fluctuations did not achieve statistical significance, the visual pattern suggests a possible stabilization or recovery of susceptibility over time. Such a trajectory is compatible with the expected effects of stewardship activities—such as more consistent diagnostic-guided therapy and refinement of empirical MRSA coverage—though no causal inference can be established from the current data. Overall, the observed decline and subsequent upward tendency may reflect underlying shifts in prescribing practices and bacterial epidemiology, warranting continued monitoring.

#### 2.2.8. *Staphylococcus epidermidis*

*Staphylococcus epidermidis* (Figure 7b) showed minimal variation in susceptibility over the study period, with consistently low percentages and no meaningful response to the January 2021 intervention. As illustrated in Figure 7b, susceptibility remained uniformly low from 2019 through 2023, with only minor fluctuations and no upward shift after ASP implementation. The Joinpoint model identified a single long segment (February 2019–September 2023) with a modest negative trend (MPC = −6.37%/month), which was not statistically significant and reflects the inherent difficulty of modifying susceptibility patterns in this organism. Given its well-known ability to form biofilms and its intrinsic resistance characteristics, *S. epidermidis* often demonstrates limited responsiveness to stewardship-driven changes in antimicrobial exposure. Overall, the temporal pattern indicates stability rather than improvement, suggesting that the ASP had little measurable impact on the susceptibility profile of this species.

#### 2.2.9. *Enterococcus faecalis*

*Enterococcus faecalis* (Figure 7c) showed a generally stable pattern with a gradual upward tendency in susceptibility across the study period. The Joinpoint analysis identified a single long segment (February 2019–September 2023) with a small, non-significant positive trend (MPC = +0.33%/month). Although this increase is modest and lacks statistical significance, the visual trajectory suggests that susceptibility values became progressively higher and more stable over time, particularly after 2021. While these observations are compatible with improvements in antimicrobial practice, the available data do not permit causal inference, and the trend should be interpreted with caution. Continued surveillance will help clarify whether this apparent upward tendency reflects a sustained epidemiological shift.

#### 2.2.10. *Enterococcus faecium*

*Enterococcus faecium* (Figure 7d) exhibited a highly variable susceptibility pattern throughout the study period, with fluctuations ranging widely between months and no discernible upward or downward trajectory, as shown in Figure 7d. The Joinpoint model identified a single long segment (December 2019–November 2023) with a non-significant declining trend (MPC = −1.77%/month), but the visual pattern reveals instability rather than a true directional change. This near-zero net variation over 53 months indicates that the ASP did not exert a visible or sustainable impact on this pathogen, a finding consistent with the biological characteristics of *Enterococcus faecium*, which is frequently multidrug-resistant and strongly associated with healthcare-associated infections, and in which resistance to last-line agents such as vancomycin (VRE), linezolid, and daptomycin may occur through acquired mechanisms [25]. The persistent variability, absence of improvement, and inherent resistance mechanisms collectively suggest that *E. faecium* requires strategies beyond routine stewardship measures, such as stricter contact isolation, active surveillance cultures, and absolute restriction of high-risk antibiotics, to meaningfully alter its resistance profile.

#### 2.2.11. All Gram-Positive Bacteria

When evaluated collectively, the Gram-positive bacteria (Figure 7e) demonstrated a complex but informative temporal pattern, marked by an initial period of stability followed by a sharp decline and a subsequent, sustained recovery. As shown in Figure 7e, susceptibility remained nearly unchanged from January 2019 to December 2021 (MPC = −0.2%/month, non-significant), indicating a stable pre-intervention baseline. A pronounced and statistically significant drop occurred between January 2022 and April 2022 (MPC = −53.75%/month; *p* < 0.05), reflecting a short-lived but abrupt deterioration. This decline was rapidly reversed in the following segment (May 2022–December 2023), which showed a strong and statistically significant improvement in susceptibility (MPC = +18.93%/month; *p* < 0.05). The resulting U-shaped trajectory suggests that although Gram-positive organisms experienced a transient disruption in susceptibility, the overall effect across the study period was a robust recovery and consolidation of higher susceptibility levels. This pattern highlights the dynamic response of Gram-positive pathogens to both ecological pressures and stewardship measures, indicating that the hospital environment, once stabilized after the transient decline favored a progressive return to a more susceptible microbial profile across the major Gram-positive species. The statistical significance observed in the aggregated Gram-positive analysis is attributable to increased statistical power and reduced variability achieved by combining multiple species. Individual species showed higher temporal variability and smaller denominators, limiting trend detection at the species level. Thus, the aggregated result reflects a system-level pattern rather than an analytical inconsistency.

#### 2.2.12. All Bacteria

The aggregated susceptibility curve of all tested bacteria (Figure 7f) provides a comprehensive view of the hospital’s microbial dynamics across the five-year study period. Between January 2019 and January 2021, susceptibility exhibited a mild but non-significant decline (MPC = −0.46%/month), reflecting a period of critical stability in which resistance pressures were present but not yet escalating sharply. This equilibrium was disrupted in the months immediately preceding the formal implementation of the ASP, when a pronounced and statistically significant deterioration emerged (January 2021–April 2021, MPC = −14.57%/month). The sharp downward inflection suggests that, by early 2021, the hospital was experiencing a measurable intensification of antimicrobial resistance across multiple pathogens, consistent with a pre-intervention environment marked by selective pressure, high antibiotic consumption, and ecological imbalance.

Following the ASP implementation, however, the overall trend reversed dramatically, demonstrating the program’s effectiveness at a system-wide level. A statistically significant improvement in global susceptibility was observed from May 2021 to June 2022 (MPC = +4.35%/month), indicating rapid ecological recovery and reduced resistance pressure in response to structured stewardship activities. In the final segment (June 2022–December 2023), susceptibility stabilized with a slight, non-significant upward slope (MPC = +0.11%/month), a pattern characteristic of mature antimicrobial stewardship systems that succeed in consolidating earlier gains while maintaining rational prescribing practices. This trajectory mirrors findings from large international evaluations, including the multicenter synthesis by Durazzi et al. (2023) [26], which demonstrated that robust stewardship programs can both reverse critical resistance trends and sustain improvements over time. The hospital’s ability to prevent regression despite ongoing clinical pressures highlights not only the immediate impact but also the long-term sustainability of its ASP, an especially relevant achievement in the global context of the escalating “silent pandemic” of antimicrobial resistance.

#### 2.2.13. Antimicrobial Consumption Savings 2019–2023

The economic analysis following ASP implementation shows a substantial reduction in antimicrobial expenditures, driven primarily by decreased consumption of high-cost agents. As presented in Table 2, all antibiotics except polymyxin B demonstrated meaningful declines in DDD/1000 patient-days, resulting in positive financial returns. Vancomycin generated the greatest projected annual savings, totaling USD 60,796.45, with a daily reduction of USD 166.57, reflecting both the high unit cost and the significant decrease in use (from 13.97 to 8.88 DDD). Meropenem and piperacillin–tazobactam also contributed prominently to overall savings, with annual reductions of USD 51,691.65 and USD 45,251.67, respectively, consistent with their role as broad-spectrum agents frequently targeted in stewardship optimization. Ceftriaxone, while a lower-cost antibiotic, still produced an annual reduction of USD 22,727.43, demonstrating the cumulative financial relevance of rationalizing high-volume antimicrobial therapy across hospital services.

Polymyxin B was the only agent to show increased consumption after ASP implementation, reflected by a negative annual balance of USD −6035.79, attributable to its exclusive use in treating multidrug-resistant Gram-negative infections, clinical scenarios in which stewardship interventions have limited ability to reduce use. Even with this increase, the aggregated economic effect remains strongly positive. The total projected annual savings reached USD 174,431.42, underscoring the financial impact of structured antimicrobial oversight. These findings confirm that the ASP not only improved prescribing practices and contributed to favorable microbiological trends but also functioned as a cost-saving strategy capable of substantially reducing direct pharmaceutical expenditures and supporting long-term institutional sustainability.

## 3. Discussion

The implementation of a structured Antimicrobial Stewardship Program (ASP) in a high-complexity public hospital resulted in substantial clinical, microbiological, and economic benefits, demonstrating the effectiveness of coordinated stewardship interventions in real-world ICU settings [8]. The significant reductions observed in broad-spectrum antibiotics, particularly ceftriaxone, meropenem, piperacillin–tazobactam, and vancomycin, confirm that prospective audit and feedback, standardized treatment protocols, and prescriber education can effectively reshape prescribing behavior in accordance with IDSA/SHEA stewardship recommendations [8]. These findings are consistent with international evidence showing that multifaceted stewardship programs reduce broad-spectrum antimicrobial consumption by approximately 20–30% in hospital settings [27,28].

At the aggregated Anatomical Therapeutic Chemical (ATC) J01 level, total antibacterial consumption decreased by 32.65% (from 410.24 to 276.30 Defined Daily Doses per 1000 patient-days) after ASP implementation, confirming that the observed impact was not limited to selected agents but reflected a true reduction in overall antibacterial exposure. Comparable reductions in total antibiotic consumption expressed as DDD per 1000 patient-days have been reported in systematic reviews and meta-analyses evaluating hospital-based stewardship interventions [27,28,29,30]. The meta-analysis by Baur et al. demonstrated that stewardship programs are associated with significant reductions in antimicrobial use and resistant organism incidence across hospital settings, supporting the ecological relevance of aggregate reductions such as those observed in our ICU [27]. Similarly, Karanika et al. reported consistent decreases in antimicrobial consumption following stewardship implementation when measured using standardized metrics such as DDD per 1000 patient-days [30].

At the substance level, reductions in meropenem (−26%), piperacillin–tazobactam (−21%), ceftriaxone (−35%), and vancomycin (−36%) align with multicenter European data demonstrating preferential decreases in broad-spectrum and Watch-group antibiotics after stewardship implementation [27,31,32]. Surveillance analyses from Polish hospitals documented measurable declines in high-impact β-lactams and carbapenems following structured stewardship oversight, a pattern comparable to our findings [31]. Likewise, multicenter studies conducted in French hospitals reported shifts in antibiotic consumption profiles, particularly affecting broad-spectrum agents under stewardship regulation [32]. These concordant findings reinforce that the reductions observed in our ICU reflect targeted ecological modulation rather than simple redistribution between agents [27,31].

From an ecological perspective, the ASP was associated with meaningful improvements in susceptibility profiles among clinically relevant Gram-negative organisms. Although intrinsically resistant pathogens such as *Enterococcus faecium* and *Acinetobacter baumannii* demonstrated limited responsiveness, *Klebsiella pneumoniae*, *Escherichia coli*, and *Enterobacter cloacae* exhibited statistically significant post-intervention improvements in susceptibility. These findings are consistent with global evidence demonstrating that reduction in broad-spectrum β-lactam pressure can facilitate restoration of susceptibility in Enterobacterales populations [4,19]. The consistent improvement observed across the Gram-negative group supports the concept that structured stewardship initiatives exert system-wide ecological effects at the hospital level [26,33].

The economic evaluation further confirmed the sustainability impact of the ASP. The projected annual savings of approximately USD 174,000, primarily driven by reductions in meropenem, piperacillin–tazobactam, and vancomycin use, demonstrate that stewardship interventions generate significant financial benefits alongside microbiological gains. These findings are consistent with international pharmacoeconomic analyses reporting reductions in antimicrobial expenditure ranging from 2% to 95% following ASP implementation [29,30]. Similar cost-saving effects have been documented in university hospital settings where structured stewardship strategies were shown to be both clinically effective and economically advantageous [34].

Importantly, large meta-analyses and multicenter surveillance studies have not consistently demonstrated parallel increases in antimicrobial resistance during periods of fluctuating antibiotic use, suggesting that well-structured stewardship and infection control measures may mitigate long-term ecological deterioration [31]. The sustained reduction and stabilization pattern observed in our post-2021 data is consistent with international evidence demonstrating that mature stewardship programs are capable of maintaining long-term control of antimicrobial consumption and resistance trends [31,32,33].

Finally, the isolate-based surveillance design adopted in this study prioritizes unit-level ecological dynamics rather than individual patient trajectories, which is methodologically appropriate for evaluating the longitudinal impact of stewardship interventions on hospital microbiology [26].

## 4. Materials and Methods

### 4.1. Study Design

This was a quasi-experimental pre–post-intervention study using an interrupted time-series (ITS) design to evaluate the impact of the Antimicrobial Stewardship Program (ASP), formally initiated in January 2021, at a public tertiary hospital in the State of São Paulo, Brazil. All data were collected from the adult intensive care unit (ICU), a high-complexity setting with substantial antimicrobial use. The pre-intervention period spanned January 2019 to December 2020, and the post-intervention period covered January 2021 to December 2023. Primary outcomes included antimicrobial consumption expressed as Defined Daily Doses per 1000 patient-days (DDD/1000 patient-days) [15], antimicrobial-related costs, and longitudinal trends in the susceptibility profiles of major clinical pathogens.

The study was conducted in a large public hospital fully integrated into the Brazilian Unified Health System (SUS) and administered under the state health authority. The ASP was structured in late 2020 and formally implemented in January 2021. The program promotes evidence-based antimicrobial prescribing, including agent selection, dosage, dosing interval, route of administration, and treatment duration, following updated national guidelines [13]. All antimicrobial prescriptions were required to include complete clinical and microbiological justification in the electronic medical record. The study population included all adult ICU patients who received systemic antimicrobials during hospitalization, and all antimicrobial use from initiation to discharge was included in the analysis. Microbiological analyses were performed at the isolate level, and all bacterial isolates recovered from these patients during ICU admission were included, regardless of repetition.

Inclusion criteria: Adult patients (≥18 years) admitted to the ICU between January 2019 and December 2023 received at least one systemic antimicrobial agent.

Exclusion criteria: Pediatric patients; outpatient or emergency department-only encounters; patients receiving only topical or non-systemic antimicrobials.

Temporal trends were evaluated using Joinpoint regression analysis, allowing identification of statistically significant inflection points in longitudinal data without predefined interruption parameters.

### 4.2. Analysis of Antimicrobial Consumption (DDD)

Antimicrobial consumption data were extracted from the SOUL MV^®^ electronic hospital information system [35] (MV Informática Nordeste Ltda, Recife, Brasil). Consumption was calculated according to the World Health Organization’s standardized metric, Defined Daily Doses per 1000 patient-days (DDD/1000 patient-days) [15]. Comparative analyses were performed between the pre-ASP period (2019–2020) and the post-ASP period (2021–2023). The five most frequently used antimicrobials: ceftriaxone, meropenem, piperacillin–tazobactam, vancomycin, and polymyxin B were selected for detailed evaluation, including monthly averages and percentage variation in consumption between the two periods.

### 4.3. Microbiological Analyses

Microbiological data were obtained from institutional laboratory spreadsheets containing 53,974 clinical cultures processed between January 2019 and December 2023. All analyses followed the infection control protocols of the hospital. Specimens were processed by an accredited outsourced microbiology laboratory using interpretative standards from the Brazilian Committee on Antimicrobial Susceptibility Testing (BrCAST) [36].

Each isolate was tested against the full panel of antimicrobials recommended by BrCAST. Therapeutic decisions followed institutional first-line protocols. For *Staphylococcus aureus*, oxacillin was used for susceptible isolates. Resistant isolates were classified as MRSA and managed with vancomycin and contact precautions. Similarly, *Klebsiella pneumoniae* susceptible to meropenem received standard treatment, whereas carbapenem-resistant isolates were classified as KPC producers and managed with contact precautions and treatment guided by the lowest minimum inhibitory concentration (MIC) among active antimicrobials.

All clinical isolates obtained from ICU patients during the study period were included in the analysis, including repeated isolates from the same patient. The unit of analysis was the isolate rather than the individual patient, in order to reflect real-world microbiological surveillance and unit-level antimicrobial resistance trends.

Surveillance and screening samples, when identifiable, were not included in the antimicrobial susceptibility analysis. Only isolates considered clinically relevant by the attending team and documented in the electronic medical record as associated with suspected or confirmed infection were analyzed. Samples deemed non-representative or clinically irrelevant were excluded whenever possible to minimize bias in AST results.

### 4.4. Economic Analysis of Antimicrobial Consumption

A retrospective economic evaluation was performed to assess the impact of the ASP on antimicrobial-related costs from January 2019 to December 2023. Consumption values (DDD/1000 patient-days) were combined with cost data based on the daily cost per DDD, using average acquisition prices obtained from the hospital purchasing department. Analyses focused on ceftriaxone, meropenem, piperacillin–tazobactam, vancomycin, and polymyxin B. For each antimicrobial, monthly consumption and percentage variation between pre- and post-ASP periods were calculated. Antimicrobial consumption was consistently expressed as Defined Daily Doses per 1000 patient-days (DDD/1000 patient-days). When mean values are reported, they refer to the average monthly DDD/1000 patient-days. Antibiotic consumption was calculated using hospital pharmacy dispensing data as the numerator and total ICU patient-days as the denominator, in accordance with the World Health Organization (WHO) ATC/DDD methodology. The WHO ATC/DDD Index version 2023 was used for all calculations.

Daily cost reductions and annual projected savings were estimated based on observed decreases in DDD/1000 patient-days. Data management and descriptive analysis were conducted using Microsoft Excel^®^ 365.

### 4.5. Statistical Analysis

Two statistical strategies were employed to evaluate the impact of the intervention on antimicrobial consumption and bacterial susceptibility.

Analysis of antimicrobial consumption: Joinpoint regression was applied using the Joinpoint^®^ software (Version 5.4.0.0) to estimate monthly percent change (MPC) and 95% confidence intervals. Between-period comparisons were performed using Student’s *t*-test or Mann–Whitney U test, as appropriate, using GraphPad InStat software, version 3.05.

Analysis of bacterial susceptibility: Only Joinpoint regression was applied [37], using aggregated monthly or quarterly susceptibility percentages.

For susceptibility assessment, each microorganism was evaluated against the institutional first-line antimicrobial agent as defined by hospital treatment protocols and interpreted according to BrCAST [36]. Carbapenems were considered first-line therapy only for severe infections and for cases with high risk or confirmed presence of extended-spectrum β-lactamase-producing or multidrug-resistant strains of *Acinetobacter baumannii*, *Klebsiella pneumoniae*, *Pseudomonas aeruginosa*, and *Enterobacter cloacae*; oxacillin for *Staphylococcus aureus* and *Staphylococcus epidermidis*; ceftriaxone for *Escherichia coli*; and vancomycin for *Enterococcus faecalis* and *Enterococcus faecium*.

### 4.6. The Implemented Antimicrobial Stewardship Program (ASP) 

The Antimicrobial Stewardship Program (ASP) implemented at our institution was based on internationally recognized core components and aligned with published hospital stewardship frameworks. Key interventions included the development and dissemination of local evidence-based treatment guidelines, prospective audit and feedback of antimicrobial prescriptions, and formulary restriction with mandatory pre-authorization for selected broad-spectrum and high-impact antibiotics.

Additional stewardship measures comprised regular multidisciplinary meetings involving infectious diseases specialists, clinical pharmacists, microbiologists, and prescribers; continuous education activities; and systematic monitoring of antimicrobial consumption and resistance trends. These interventions are consistent with those described in previous European hospital stewardship studies and were progressively reinforced during the study period [31,33].

## 5. Conclusions

In conclusion, this study provides robust real-world evidence that a structured ASP can meaningfully transform antimicrobial use, improve susceptibility patterns, and reduce healthcare costs in a public tertiary hospital in Brazil. The program successfully achieved its primary objectives: reducing unnecessary exposure to broad-spectrum antibiotics, mitigating selective pressure on the hospital eclogy, and promoting consistent and guideline-adherent prescribing practices. The ecological gains observed, particularly in key Gram-negative pathogens and the substantial economic impact underscore the importance of institutionalizing ASPs as permanent healthcare policies. In the context of the escalating global “silent pandemic” of antimicrobial resistance, this study contributes valuable national evidence supporting the critical role of stewardship programs in patient safety, clinical quality, and the long-term sustainability of healthcare systems.

### Study Limitations

This study has some limitations. Because isolates were not deduplicated at the patient level, repeated cultures from the same patient may have contributed to the dataset, potentially overrepresenting patients with prolonged ICU stays. However, this isolate-based approach is consistent with ecological surveillance studies and was intentionally adopted to evaluate temporal trends in antimicrobial resistance at the ICU level rather than patient-level outcomes. Second, although efforts were made to focus on clinically relevant isolates, the retrospective design limited the ability to uniformly exclude surveillance or screening samples across all periods. These factors may have influenced antimicrobial susceptibility distributions and should be considered when interpreting the results. Finally, as this was a single-center observational study, unmeasured confounders related to case-mix and clinical practice changes cannot be entirely excluded.

## Figures and Tables

**Table 1:** Distribution of the most frequently isolated bacterial species in the adult intensive care unit between January 2019 and December 2023. Bold type indicates ESKAPE pathogens.

Microorganism	N	%
***Klebsiella pneumoniae***	2246	26.23
***Staphylococcus aureus***	1053	12.30
*Escherichia coli*	1025	11.97
***Pseudomonas aeruginosa***	768	8.97
***Enterococcus faecalis***	614	7.82
*Staphylococcus epidermidis*	536	6.26
***Acinetobacter baumannii***	459	5.36
***Enterobacter cloacae***	229	2.67
*Enterococcus faecium*	224	2.62
*Proteus mirabilis*	207	2.42
*Serratia marcescens*	179	2.09
*Staphylococcus haemolyticus*	154	1.80
*Stenotrophomonas maltophilia*	134	1.56
*Staphylococcus capitis*	117	1.37
*Staphylococcus hominis*	102	1.19
*Morganella morganii*	94	1.10
*Klebsiella oxytoca*	83	0.97
*Klebsiella aerogenes*	70	0.82
*Streptococcus pneumoniae*	61	0.71
*Citrobacter koseri*	37	0.43
*Citrobacter freundii*	32	0.37
*Haemophilus influenzae*	27	0.32
*Klebsiella variicola*	26	0.30
*Enterobacter asburiae*	26	0.30
*Enterobacter bugandensis*	23	0.27
*Aeromonas hydrophila*	21	0.25
*Burkholderia cepacian*	18	0.21

**Table 2:** Reduction in consumption and daily and annual antibiotic costs (in US$) after ASP implementation.

Antibiotic	DDD Before ASP	DDD After ASP	Reduction	DDD Cost (US$)	Daily Cost Reduction (US$)	Annual Reduction (US$)
Ceftriaxone	22.93	14.73	8.19	1.40	62.27	22,727.43
Meropenem	9.00	6.61	2.38	10.92	141.62	51,691.65
Piperacillin + tazobactam	11.24	8.85	2.39	9.52	123.98	45,251.67
Vancomycin	13.97	8.88	5.09	6.01	166.57	60,796.45
Polymyxin B	2.77	3.72	−0.95	3.20	−16.54	−6035.79
Total						174,431.42

DDD corresponds to Defined Daily Doses per 1000 patient-days, calculated using ICU patient-days as the denominator, according to the WHO ATC/DDD Index.

## Data Availability

The data supporting the findings of this study are available from the authors upon reasonable request. Interested researchers may obtain the data by contacting the corresponding author.
